# VDJtools: Unifying Post-analysis of T Cell Receptor Repertoires

**DOI:** 10.1371/journal.pcbi.1004503

**Published:** 2015-11-25

**Authors:** Mikhail Shugay, Dmitriy V. Bagaev, Maria A. Turchaninova, Dmitriy A. Bolotin, Olga V. Britanova, Ekaterina V. Putintseva, Mikhail V. Pogorelyy, Vadim I. Nazarov, Ivan V. Zvyagin, Vitalina I. Kirgizova, Kirill I. Kirgizov, Elena V. Skorobogatova, Dmitriy M. Chudakov

**Affiliations:** 1 Shemyakin-Ovchinnikov Institute of bioorganic chemistry RAS, Moscow, Russia; 2 Pirogov Russian National Research Medical University, Moscow, Russia; 3 Central European Institute of Technology, Masaryk University, Brno, Czech Republic; 4 National Research University Higher School of Economics, Moscow, Russia; 5 Russian Children's Hospital, Moscow, Russia; University of Canterbury, NEW ZEALAND

## Abstract

Despite the growing number of immune repertoire sequencing studies, the field still lacks software for analysis and comprehension of this high-dimensional data. Here we report VDJtools, a complementary software suite that solves a wide range of T cell receptor (TCR) repertoires post-analysis tasks, provides a detailed tabular output and publication-ready graphics, and is built on top of a flexible API. Using TCR datasets for a large cohort of unrelated healthy donors, twins, and multiple sclerosis patients we demonstrate that VDJtools greatly facilitates the analysis and leads to sound biological conclusions. VDJtools software and documentation are available at https://github.com/mikessh/vdjtools.

## Author Summary

High-throughput profiling of T- and B-cell antigen receptor repertoires promises great advances in our understanding of the mechanisms underlying adaptive immune system function, treatment of autoimmune and infectious diseases, and development of novel approaches in cancer immunotherapy. A number of recently developed software tools aim at processing immune repertoire data by mapping Variable (V), Diversity (D) and Joining (J) antigen receptor segments to sequencing reads and assembling T- and B-cell clonotypes. Nevertheless, there still exists a major gap in common methods of data post-analysis in the field: there is no standardized data format so far, and most of data comparative analysis is carried out using a variety of in-house scripts. Here we present VDJtools, a software framework that can analyze output of most commonly used TCR repertoire processing tools and allows applying a diverse set of post-analysis strategies. The main aims of our framework are: To ensure consistency of post-analysis methods and reproducibility of obtained results; to save the time of bioinformaticians analyzing TCR repertoire data by providing comprehensive tabular output and open-source API; and to provide a simple enough command line tool so that immunologists and biologists with little computational background could use it to generate publication-ready results.

This is a *PLOS Computational Biology* Software paper

## Introduction

The advent of high throughput sequencing (HTS) has opened a new venue for the studies of genomics of adaptive immunity that involve deep profiling of T-cell receptor (TCR) and B-cell receptor (BCR) gene repertoires encoding a myriad of antigen specificities.

Huge volumes of complex data produced by the immune repertoire profiling have led to the development of a diverse set of software tools, which often complement each other. We [1–3] and others [4–7] have recently contributed several tools that handle large amounts of raw HTS data to process it into a human-readable list of clonotypes characterized by Variable (V), Diversity (D), Joining (J) segments and V-(D)-J junction sequences of receptor genes. While such processed data carry nearly exhaustive information on the sampled immune repertoire, this information yet needs to be convolved, scaled and compared across various samples to result in sound biological conclusions.

Post-analysis of immune repertoire data is a challenging task owing to extreme diversity of TCR and BCR sequences. For example, in technically similar microbiome profiling by 16S rRNA sequencing one deals with thousands of operational taxonomic units that represent various species [8], while typical TCR repertoire samples may contain hundreds of thousands [9,10] of clonotypes. Moreover, the species phylogeny and annotation is well developed in the field of microbiology [11], while immune repertoires remain poorly annotated. To illustrate this, a simple query with “16S rRNA” currently yields more than 8 million records in GenBank, while there are only 37 thousand records annotated as “T-cell receptor”. However, unsupervised methods of studying repertoires, for example based on sample overlap, could turn out very promising, as there exists a relatively limited diversity of overlapping clonotypes [12–15].

In the light of recent advances in storage and processing of immunological big data [16], community-driven initiatives for immune repertoire data sharing and analysis are likely to emerge, for example VDJserver portal [17] which is currently under development. There are several commonly used ways to survey immune repertoire information obtained from HTS, such as tracking individual clonotypes [18,19], comparing immune receptor segment usage [20,21] and comparing repertoire diversity [10]. Still those are overwhelmingly performed using in-house scripts or even manually. This is becoming a major obstacle, as comparison and annotation of samples based on data generated in other studies is critical for comprehensive analysis of immune repertoire sequencing data. In contrast, similar fields, such as metagenomics, have a plethora of such instruments [22].

**Fig 1:**
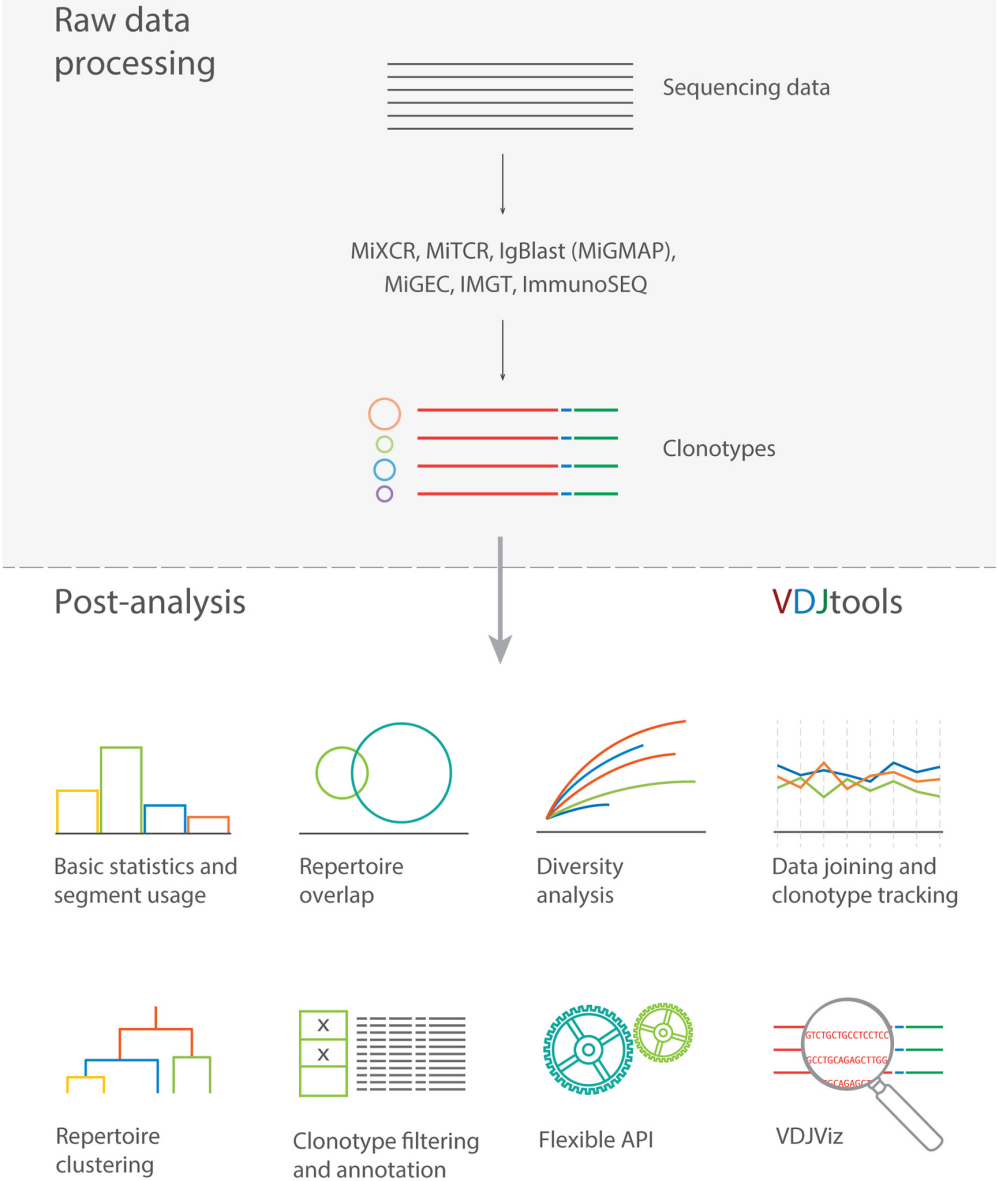
Overview of VDJtools software package. VDJtools analysis routines can be grouped into 6 modules and are applicable to results produced by commonly used immune repertoire sequencing processing software. Basic statistics and segment usage module include general statistics (clonotype and read count, number and frequency of non-coding clonotypes, convergent recombination of CDR3 amino acid sequences, insert size statistics, etc), spectratyping (distribution of clonotype frequency by CDR3 length), Variable and Joining segment usage profiles and their pairing frequency in re-arranged receptor junction sequences. Repertoire overlap module includes routines for computing sets of overlapping clonotypes and their characteristics, and scatter plots of clonotype frequencies. Diversity analysis includes routines for visualizing clonotype frequency distribution, computing repertoire diversity estimates and rarefaction plots. The fourth set of routines can be used to create clonotype abundance profiles and track clonotypes in time course of vaccination, myeloablation and blood cell transplant. Sample clustering is implemented based on computed repertoire similarity measures and could be used to distinguish various biological conditions, cell subsets and tissues. Auxiliary routines provide means for clonotype table filtering (e.g. by segment usage or non-coding CDR3 sequence) as well as annotation with custom or pre-built pathogen-specific clonotype database. VDJtools can be incorporated in Java programming language-based pipelines as demonstrated by VDJviz clonotype browser.

The VDJtools software package presented here aims at filling this gap by incorporating a comprehensive set of routines for analysis of TCR repertoire sequencing data (Fig 1). The variety of implemented algorithms range from basic statistics calculation and clonotype table filtering to advanced routines such as repertoire clustering and computationally intensive routines such as clonotype table joining.

VDJtools can calculate basic immune repertoire statistics that were commonly used in pre-HTS era repertoire analysis. Those include *in silico* spectratype (the distribution of lengths of CDR3 nucleotide sequences) that was first introduced with corresponding molecular biology assay [23], and various Variable/Joining segment usage statistics that root in flow cytometry analysis of T- and B-cell populations.

The framework provides means for analyzing the diversity of immune repertoires, such as normalized unique clonotype counts (with an option to account for convergent recombination), clonotype frequency distribution, as well as rarefaction curves and lower bound estimates of total repertoire diversity widely applied in ecology field [24]. The concept of repertoire diversity is of great importance, as it reflects the ability of our immune system to effectively withstand a multitude of encountered pathogens [25]. By applying computational methods one could estimate how the diversity is influenced by various processes, such as aging [10], vaccination, and infection [26]. Diversity measures could also be used to compare the structure of T- and B-cell repertoires in samples derived from a variety of tissues and subjects [27].

Advanced set of VDJtools methods includes cluster analysis of repertoire samples and clonotype tracking which have a wide range of applications. Machine learning methods such as hierarchical clusterization and multi-dimensional scaling can aid in learning T-cell antigen specificities and disease biomarker patterns from high-dimensional TCR data [28]. Clonotype tracking is useful in studying immune repertoire dynamics associated with vaccination [29], autologous hematopoietic stem cell transplantation (HSCT) [19,30,31], checkpoint inhibitors [32], etc., as well as in detection of minimal residual disease in lymphoid malignancies [33–37].

An overview of 20 recently published immune repertoire studies (S1 Table) demonstrates that VDJtools can perform most of emerging post-analysis tasks therefore greatly facilitating the analysis process and removing the need to develop multiple custom scripts. Currently there are few software tools capable to perform post-analysis of immune repertoire data [7,38,39], all of which provide less functionality when compared to VDJtools (S2 Table). Moreover, in contrast to VDJtools which can handle output generated by various pre-processing software, these tools only support datasets in their internal formats.

## Design and Implementation

The study was approved by ethics committee of the Russian Children's Hospital from January 20, 2011.

The core API of the software is implemented in Java/Groovy languages and automatically resolves all dependencies during compilation using Maven. The API includes generalized entities, such as *Clonotype*, *Sample* and *SampleCollection* classes, and allows storing sample metadata using *MetadataTable* class. The API also contains a comprehensive set of routines for computing sample-specific and cross-sample statistics, which are optimized for parallel computation. VDJtools API can be easily integrated in any software written in Java or related programming languages (e.g. Groovy, Scala and Clojure). VDJtools is an open-source software, the source code can be accessed at GitHub [40].

Comprehensive software documentation is hosted at ReadTheDocs [41] and contains basic usage guidelines (including the description of common pitfalls), a summary of implemented algorithms, as well as examples that cover some typical VDJtools usage cases. The documentation also contains step-by-step instructions for reproducing the analysis described in present paper.

VDJtools has a command line interface that allows executing analysis routines that produce tabular and publication-ready graphical output. Tabular output can be used for post-hoc analysis in R or explored in spreadsheet software such as Excel. Plotting parameters are optimized to provide the most intuitive and comprehensive graphical representation for most usage cases while users can specify their own sample groups and factors to be visualized.

VDJtools accepts tabular output of commonly used pre-processing software: MIGEC [2], MiTCR [1], ImmunoSEQ [38], IMGT/HighV-QUEST [4], and MiXCR [3]. VDJtools also supports IgBlast [5] software format. Of note, using IgBlast requires a considerable amount of parsing and post processing, as it only reports Variable segment alignment and doesn’t provide the CDR3 sequence. Moreover, vanilla IgBlast doesn’t accept FASTQ format input, does not provide clonotype assembling (grouping of sequencing reads with identical Variable segment, Joining segment and CDR3 sequence) and is not optimized for parallel computations. We have implemented all those features in our wrapper for IgBlast software, MIGMAP, that could be downloaded from [42]. VDJtools converts all input datasets to its own internal format, which is a tab-delimited table containing abundance, CDR3 sequence, V, D and J segment names and markup of CDR3 sequence germline regions.

An immune repertoire browser VDJviz which serves as a lightweight GUI for VDJtools was built using Play framework and VDJtools API and could be accessed at [43].

Raw data for multiple sclerosis patients is deposited at SRA (PRJNA280417). Pre-processed clonotype tables can be found in a separate GitHub repository [44], which also contains shell scripts that can be used to reproduce the analysis.

## Results

To demonstrate the efficiency of VDJtools, we have analyzed TCR beta repertoires for the peripheral blood samples of 13 young (6–15 years old) individuals diagnosed with multiple sclerosis (MS1-13), and 6–25 years old control group (C1-11) described in Ref. [10]. The multiple sclerosis dataset was prepared and sequenced using the same protocol as the control one. We have also included a sample from the MS8 patient after hematopoietic stem cell transplant (MS8HSCT). The list of samples is provided in S3 Table.

To remove quantitative biases and reduce possible impact from PCR and sequencing artifacts, we have utilized unique molecular identifiers [10,45,46]. Analysis of raw molecular barcoded data was performed using our MIGEC software. Molecular identifier groups represented by a single read were discarded, and the remaining groups were subjected to cDNA consensus assembly and CDR3 extraction as previously described [2]. Hereafter we will use the term T-cell receptor beta chain cDNA molecules (TRBM) for describing clonotype count units. Note that in these experiments we obtained ~0.5 mln cDNA molecules per ~1–10 mln starting T-cells, so we can assume that each TRBM roughly represents a single T cell.

### Estimating repertoire diversity

We have started our analysis by comparing the repertoire diversity of MS and control samples. To support the diversity measure choice and check for possible biases we have performed a benchmark on previously published T cell immunity aging data [10] and additional ANOVA analysis to identify factors that bias diversity estimates (S1 Text, S1 Fig, S4 Table). We have used common diversity measures: the observed diversity (number of unique clonotypes), Chao [47] and Efron [48] estimates for lower bound on total species diversity, Shannon [49] and Simpson [50] indices, as well as extrapolated Chao estimate [51].

**Fig 2:**
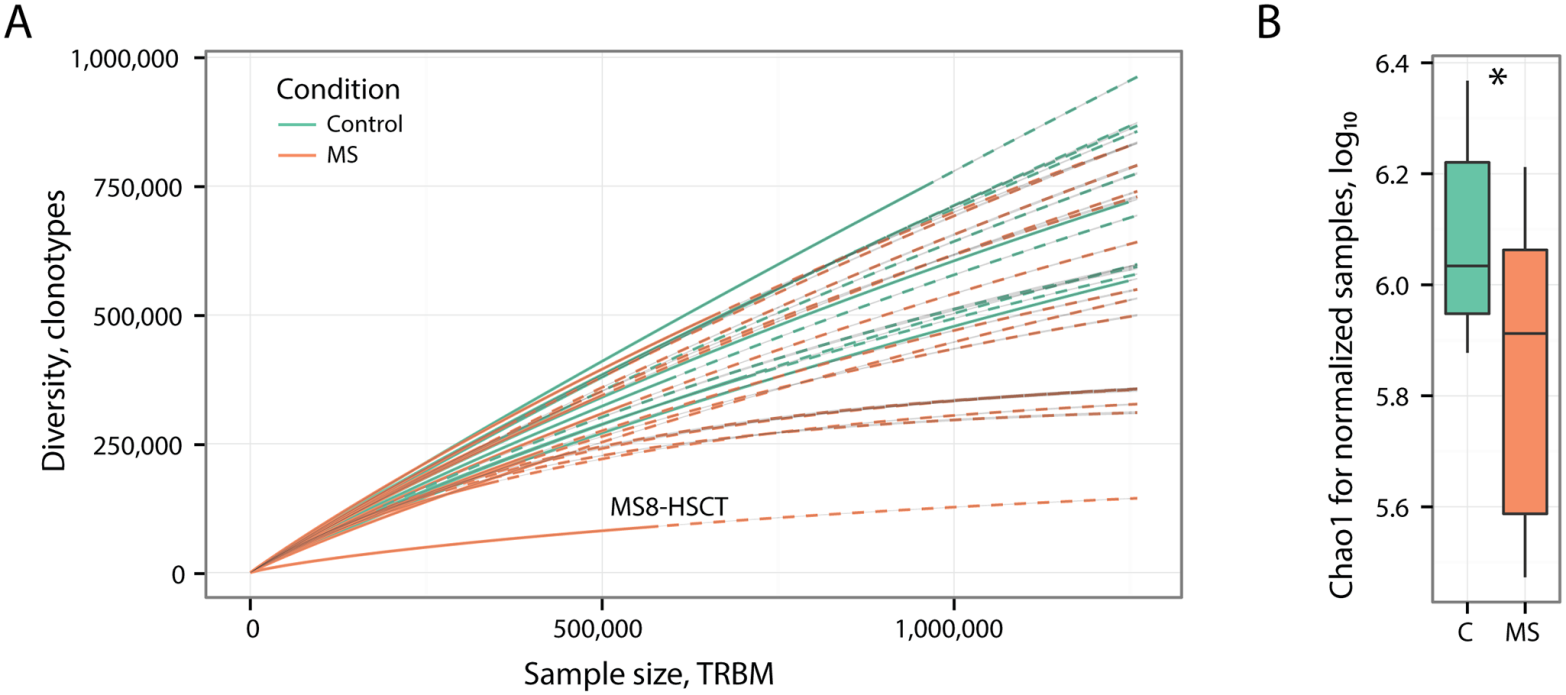
Estimation of repertoire diversity using multinomial model. **A.** Rarefaction analysis of repertoire samples from healthy donors and multiple sclerosis patients. The number of unique clonotypes in a sub-sample plotted against its size (number of T-cell receptor cDNA molecules, TRBM). Solid and dashed lines are diversity estimates computed by interpolating and extrapolating using a multinomial model respectively [29]. Note that generally rarefaction curves for MS samples go below those of control donors. Post-HSCT sample (MS8-HSCT) displays the lowest diversity. **B.** Comparison of repertoire diversity using normalized Chao1 estimate. Normalization is performed by down-sampling datasets to the size of smallest dataset and computing the estimate for resulting datasets (mean estimate value from n = 3 re-samples is used). MS8-HSCT sample is discarded from calculations. *—P = 0.022, two-tailed T-test; effect size estimated by Cohen’s d is 0.98.

The benchmark, in which correlation with a physiological (age) and immune status (naïve T-cell count) factors was compared for various diversity estimates, has shown that best correlation can be achieved when samples are normalized to the same size (TRBM count). Correspondingly, ANOVA analysis suggests a strong sampling-related bias. Accounting for this bias is especially important in present case as the rarefaction curves are far from saturation (Fig 2A). Notably, lower bound estimates of total repertoire diversity that are especially affected by sampling bias were applied in some recent studies for the comparison of TCR repertoire diversity under uneven sample sizes [9,52].

Using Chao1 estimate [47] for normalized datasets that has shown the best performance together with Efron estimate (yet is far simpler to compute) in the aforementioned benchmark, one can discover that MS samples have a significantly lower diversity than the controls (Fig 2B). This suggests a substantial expansion of T-cell clones in peripheral blood of MS patients, an observation previously supported only by local measurements such as Sanger sequencing of individual T-cells and spectratyping assays [53]. As control population is slightly older than MS group one can expect even more profound difference in case exact age matching is achieved for the control group [10]. Still, there is no significant difference for the directly observed sample diversity (S2 Fig), which is likely due to the fact that this estimate doesn’t account for the clonotype frequency distribution in sample and thus is less sensitive.

### Cluster analysis of repertoires

As there is currently no study describing an application of cluster analysis to a large set of immune repertoire datasets coming from different individuals, we have performed a benchmark of various clustering strategies using a recently published twins TCR repertoires study [54]. We have tested the ability to distinguish TCR repertoires of identical twins from those of unrelated individuals for several commonly used similarity measures, correlation of overlapping clonotype frequencies (*R*), geometric mean of total frequencies of overlapping clonotypes (*F*), normalized number of overlapping clonotypes (*D*, [14]), Jaccard [55] and Morisita-Horn indices [56]. Only the *F* similarity measure showed significant difference for both TCR alpha and beta chain datasets (S1 Text, S5 Table and S3 Fig). At the same time, it should be noted that *R* and *D* measures also proved to be useful in other experimental setups. For example, *R* measure accurately separated TCR alpha repertoires for the T cell subsets and tissues, as well as mutant and control mice Treg repertoires [57].

We have next used cluster analysis to explore whether TCR beta repertoires of MS patients can be distinguished from healthy controls. As some samples were prepared in parallel with single-end sample barcoding, joined and then co-amplified after Illumina adapter ligation, we first checked for the possibility of cross-sample contamination (S4 and S5 Figs). It turned out that direct clustering of samples with *F* measure resulted in a strong co-clustering of samples prepared in the same batch. To correct for batch effect, we have selected “amino acid NOT nucleotide” clonotype intersection matching rule, i.e. matching of CDR3 amino acid, but not the nucleotide sequences.

**Fig 3:**
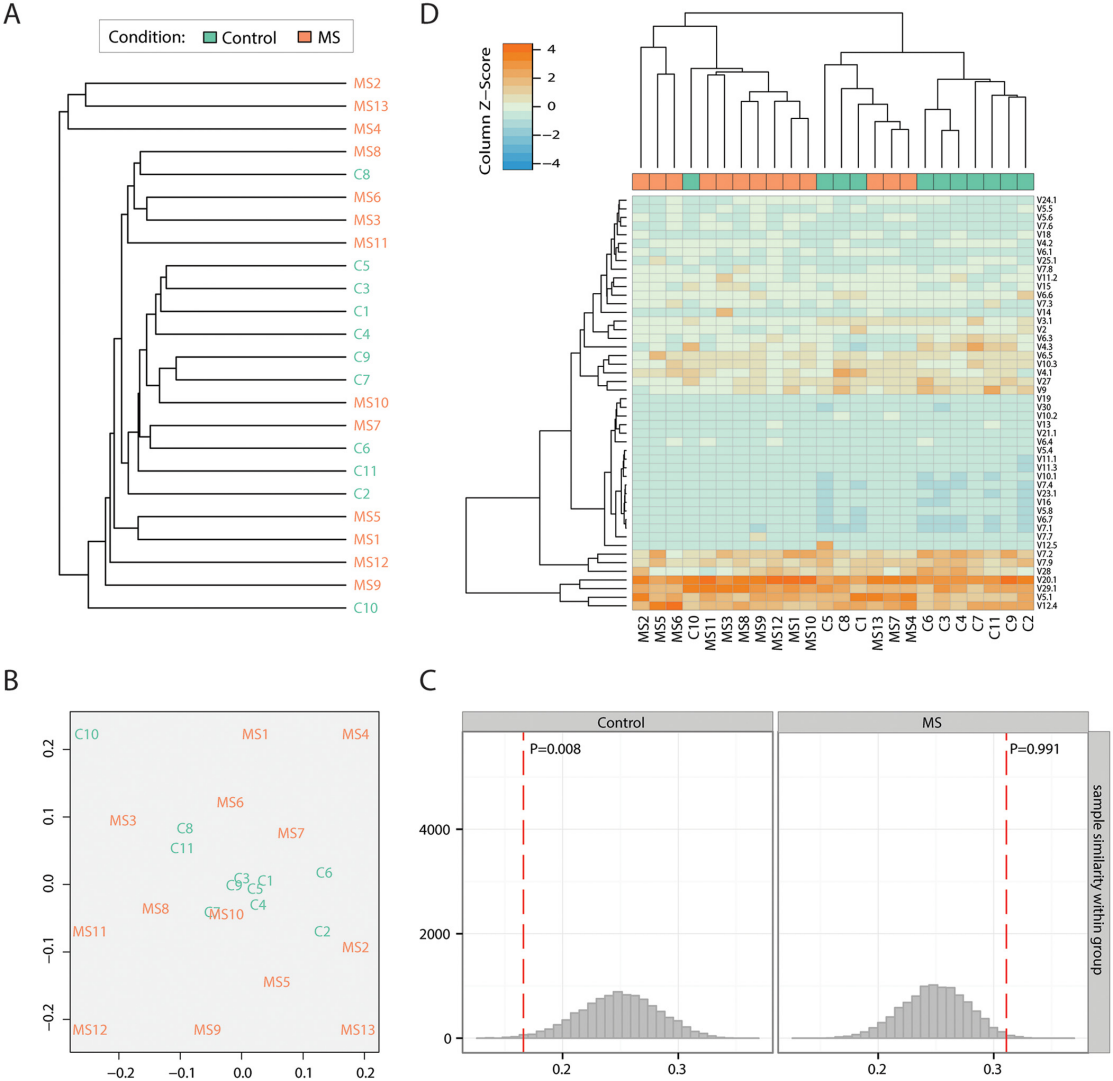
Overlap and clustering of TCR repertoires. **A.** Hierarchical clustering of healthy donor and multiple sclerosis (MS) patient samples using F pairwise similarity metric (the geometric mean of the total frequency of overlapping clonotypes in first and second sample in pair). **B.** Multi-dimensional scaling (MDS) plot. Samples were projected onto two-dimensional plane based on pairwise similarities (F metric). **C.** Permutation testing for closeness of samples coming from the same group based on MDS plot. The plot shows observed (dashed red lines) and permuted (histograms) average within-group sample distance. In contrast to control group, MS group displays highly dissimilar T-cell repertoires. N = 10,000 permutations of group labels were performed. **D.** Hierarchical clustering of samples based on the Euclidean distance between Variable segment frequency vectors. Note that the clustering provides a nice separation between sample groups (Control and MS, P = 0.013, Fisher’s exact test).

Hierarchical clustering with *F* similarity measure and “amino acid NOT nucleotide” clonotype matching rule showed some co-clustering for control but not MS datasets (Fig 3A). Further exploration with multidimensional scaling (MDS) method showed that control repertoires of healthy children are more similar to each other according to *F* similarity measure, while MS repertoires are all different (Fig 3B and 3C). This result is quite similar to our observations of age-related changes in TCR repertoires (our unpublished data). With aging, expansion of antigen-specific clones moves away native repertoires that are initially more close to each other due to the public clonotypes that are frequently produced in recombination [58]. This is in line with observation of early clonal T-cell expansions in MS children (see “Estimating repertoire diversity” section above). Since those expanding T-cell clones, including potentially autoreactive ones, are predominantly private to an MS-affected person [59–61] this leads to the decrease of the overlap between MS repertoires according to the clonotype size-weighted *F* similarity measure.

### T-cell receptor segment usage signatures

Keeping in mind that MS was shown to have a Type I-II TCR repertoire bias [62], i.e. the same prominent Variable segment is used, yet only limited homology between CDR3 region is present in disease specific T-cells, we have performed hierarchical clustering of Variable segment usage profiles (Fig 3D, note that profiles are weighted by TRBM count). The resulting dendrogram distinguishes MS patients and healthy donors with 91% sensitivity and 77% specificity (P = 0.013, Fisher’s exact test for cluster—group association).

A post-hoc testing was then performed to find out which Variable segments were more abundant in MS donors than in healthy controls (S6 Table). We have determined that 5 Variable segments had a statistically significant increase in frequency, including TRBV5-6 (1.6-fold, P = 2x10^−5^) and TRBV5-1 (1.5-fold, P = 5x10^−4^), which were previously reported to have a genetic association with MS [61,63]. Of note, TRBV20-1 (1.3-fold, P = 2x10^−3^) which has also emerged in our results was recently shown to have no genetic association with MS in a Sicilian population carrying null allele [64]. This suggests that the observed TRBV20-1—MS association could be either specific for Russian population or represent an indirect biomarker.

### Tracking repertoire changes induced by hematopoietic stem cell transplantation

**Fig 4:**
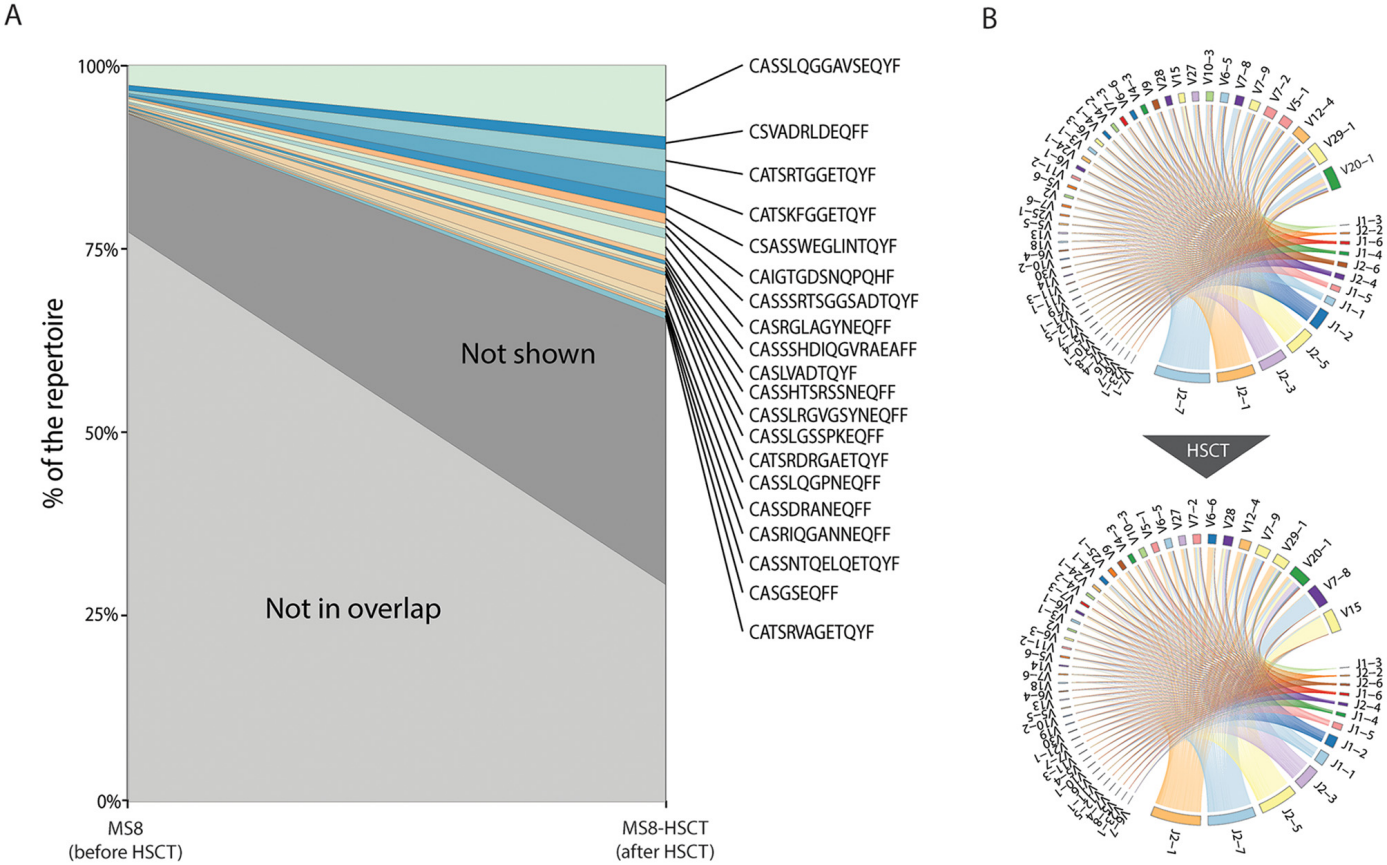
Analysis of autologous HSCT-driven changes in T-cell repertoire. **A.** Stacked clonotype frequency plot highlighting the details of overlap between sample MS8 (before autologous HSCT) and MS8-HSCT (post HSCT). Top 100 clonotypes based on their average frequency in those samples are shown, while other clonotypes that were observed in both samples are marked as “Not shown”. The frequency of remaining clonotypes is marked as “Not in overlap”. **B.** Changes in Variable-Joining segment pairing in CDR3 junctions changes induced by HSCT. Chord diagram is used for visualization, ribbons connecting segment pairs are scaled by corresponding V-J pair frequency. “TRB” prefix is stripped from segment names for simplicity.

Further we have compared TCR repertoires of blood samples taken from a single MS patient (MS8) before and after HSCT (see Fig 4). We have first tracked the clonotypes present before HSCT procedure to the post-transplantation repertoire (Fig 4A). The resulting plot clearly shows that pre-transplantation clones greatly expand (from ~25% of TRBMs to 75%) and occupy most of homeostatic space in post-HSCT repertoire. The magnitude of this effect resembles the one we previously observed in an ankylosing spondylitis patient HSCT case [19] and in adult MS autologous HSCT study [31].

Another peculiar finding is that a strong shift in Variable segment usage is observed, while no such change is present for J segment usage (Fig 4B). TRBV15 and TRBV7-8 ranking 10 and 5 replaced the top two Variable segments TRBV20-1 and TRBV29-1, while top two Joining segments TRBJ2-7 and TRBJ2-1 remained the same. This could not be attributed to CD4/CD8 balance alone, as there is strong differential Joining segment usage between those two populations [65]. Interestingly, a significant HSCT-induced decrease was observed for TRBV5-6, TRBV5-1, TRBV5-8, TRBV7-6 and TRBV20-1 (P = 0.008, two-tailed paired T-test for log TRBM frequencies) segments that were enriched in MS patients compared to healthy controls (see previous section). The total frequency of those segments dropped from 20% of TRBMs to 14%.

### Comparing bulk characteristics of CDR3 regions for MS patients and healthy donors

**Fig 5:**
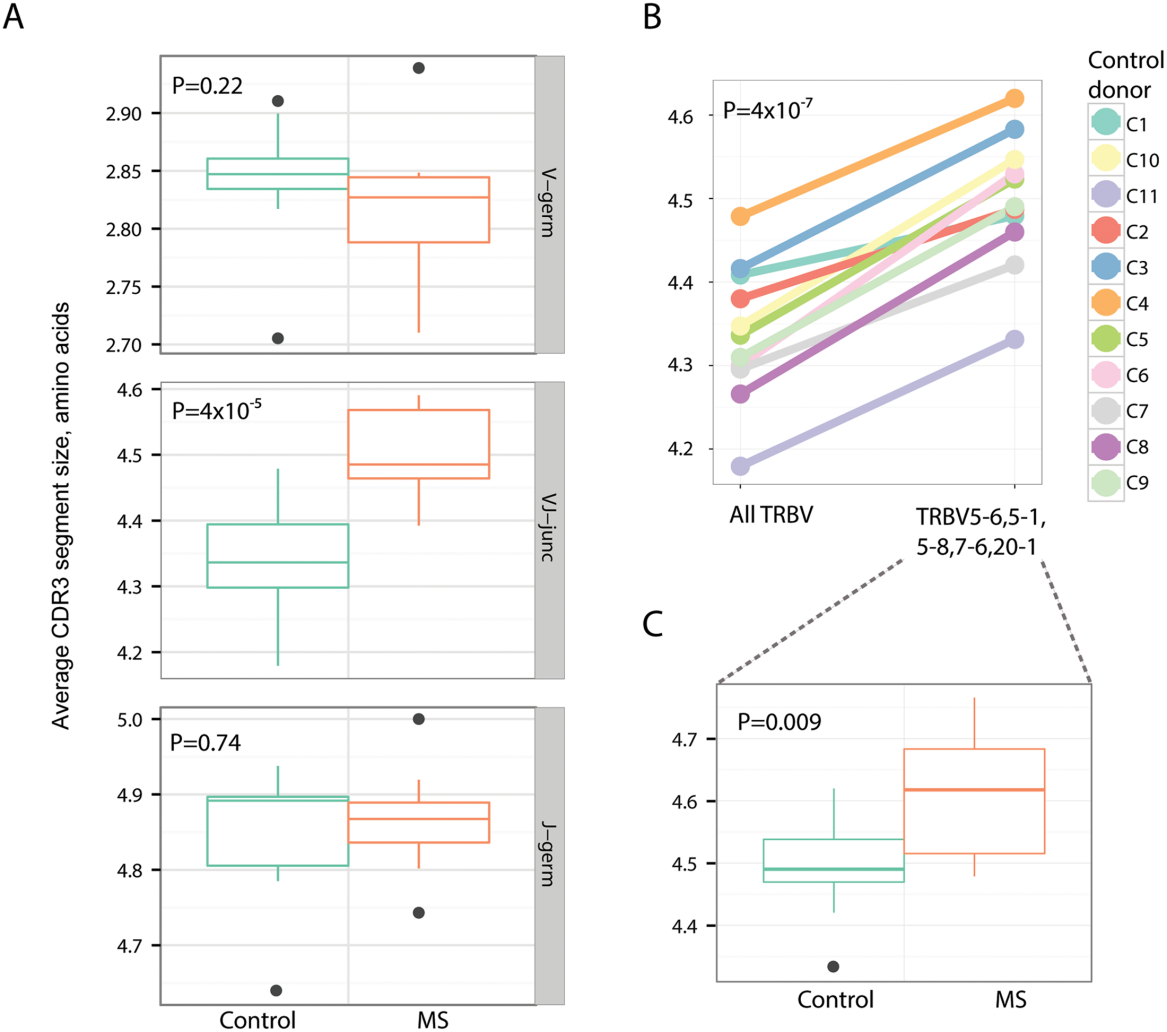
CDR3 junction features. MS patient-derived repertoire is enriched for TCR sequences with long VJ insert, partially due to high abundance of specific Variable segment regions. **A**. Length of Variable and Joining segment germline parts within CDR3 (V-germ and J-germ) and of VJ insert (VJ-junc) compared between MS donors and healthy controls. **B**. Average length of VJ junctions among all and selected V-segments (TRBV5-6,5–1,5–8,7–6 and 20–1, shown to be over-expressed in MS patients compared to controls, see main text) according to TCR sequences from repertoires of healthy donors. **C**. Comparison of VJ insert lengths between control and MS donors for clonotypes with TRBV5-6,5–1,5–8,7–6 and 20–1 segments. P-values computed using two-tailed unpaired T-test (A, C) and paired T-test (B).

Finally, we have compared CDR3 regions of MS patients to healthy donors using a set of basic features: the length of Variable and Joining segment germline parts remaining within CDR3 region, and VJ junction (NDN) size. The length of CDR3 segment itself is a potent marker of antigen receptor reactivity. For example, longer CDR3 sequences may be more characteristic for potentially cross- and self-reactive immune receptors [66], while CDR3 variants with low number of randomly added “N” nucleotides are characteristic for public clonotypes, including variants specific to common pathogens such as EBV and CMV [67]. As our analysis shows, MS patients are characterized by longer VJ junction region (Fig 5A). To check whether it is due to specific segment usage profile we have compared VJ junctions from all clonotypes of normal donors to the ones coming from clonotypes that have one of Variable segments previously shown to be over-expressed in MS patients (Fig 5B). We have found that aforementioned TRBV5-6, TRBV5-1, TRBV5-8, TRBV7-6 and TRBV20-1 are intrinsically characterized by longer VJ inserts. However, there is still a significant difference in VJ junction size between MS patients and controls for this subset of TRBV segments (Fig 5C). These results may indicate that clonal expansions in MS patients are characterized by more self-reactive T-cell clonotypes than in healthy donors. Alternatively, this could be a more general hallmark of chronic inflammation associated with MS.

## Availability and Future Directions

A cross-platform binary version of software in a form of executable JAR file is available from [68]. VDJtools software is free for scientific and non-profit use. The source code is available at GitHub repositories [40] and [69].

One important aspect of VDJtools usage not mentioned in the results section is the benchmark of pre-processing software (S6 Fig) and library preparation protocols. For this purposes we plan to constantly update VDJtools so it is able to handle the output of newly developed pre-processing software.

In future we plan extending VDJtools software to address another highly important problem in the field, the analysis of antibody repertoire [70]. While being applicable to the analysis of BCR clonotypes, VDJtools currently doesn’t account for somatic hypermutations and therefore yet cannot offer a comprehensive analysis for the antibody repertoires. This task requires us to implement algorithms for computing statistics of hypermutation transition patterns and reconstruction of B-cell clonal lineages and visualization of hypermutation graphs. We are also looking forward for the feedback from the community to meet the demand for some exciting novel features that will surely arise in this rapidly growing field.

## Supporting Information

S1 Text Description of dataset and benchmarks. (PDF)

S1 Table Overview of 20 recently published T-cell repertoire sequencing studies. Primary analysis software and post-analysis methods that are supported by VDJtools are highlighted in green. Note that none of these papers indicate using specialized software for analysis of clonotype tables, therefore post-analysis in each case was performed either manually or using in-house scripts developed from a scratch.
(DOCX)

S2 Table Comparison of VDJtools with existing software tools. This table contains summary of features present in VDJtools and other immune repertoire post-analysis software (ImmunoSEQ analyzer [38], Vidjill [7] and AbMining Toolbox [39]).
(DOCX)

S3 Table Sample metadata. Metadata for MS and control samples. Donor age, gender and condition are provided. Samples in the same batch were prepared together, multiplexed and sequenced on the same HiSeq lane.
(DOCX)

S4 Table ANOVA summary for various factors that affect the repertoire diversity estimates. Interaction between factors is shown with “:” sign.
(DOCX)

S5 Table Repertoire similarity. The ability of repertoire similarity measures to distinguish identical twins (n = 3 pairs) from unrelated individuals (n = 12) for TCR alpha and beta chain samples. Statistical significance and effect size were assessed using two-tailed T-test P-values and Cohen’s d.
(DOCX)

S6 Table Variable segments that are highly used in MS patients. In order to determine the possible Type I-II bias according to Ref. [62], i.e. sharing of common repertoire features under the absence of common clonotypes, in TCR repertoires of MS patients we have performed multiple testing for TRBV frequency difference using one-tailed T-test. One-tailed T-test was chosen to increase the power as we *a priori* search for an expansion in the T-cell compartment. Appropriate correction for multiple testing was applied (Benjamini-Hockberg correction). Variable segments that are significantly over-represented in MS samples comparing to control are shown.
(DOCX)

S1 Fig Repertoire diversity estimator performance. This plot shows Spearman correlation of diversity estimate with age and naïve T-cell count. Unmodified samples (exact) and samples normalized to the same size (resampled) from the “aging” study were used (n = 39). Note that ChaoE is omitted from the “resampled” plot, as it equals observed diversity when samples are of the same size.
(TIF)

S2 Fig Difference in repertoire diversity between Control and MS. Difference was measured using four repertoire diversity estimates considered in present study (separate panels). The effect sizes are 1.21, 0.98, 0.95 and 0.46 respectively (Cohen’s d). **—P < 0.01, *—P < 0.05, ns—non-significant, two-tailed T-test.
(TIF)

S3 Fig Similarity measures. Values of similarity measures for identical twins and unrelated individuals that were used for statistical testing in S5 Table.
(TIF)

S4 Fig Possible biases in sample clustering in present study. **A.** Hierarchical clustering of repertoires based on two distinct clonotype matching rules: matching CDR3 amino-acid sequences (**left panel**) and matching of CDR3 amino acid sequences but distinct CDR3 nucleotide sequences (**right panel**). Batch effect for samples on the same sequencing lane is shown with vertical lines. **B.** Checking for possible sex bias in repertoire clustering. Multi-dimensional scaling (MDS) plot is shown for healthy donors of various ages and sexes from the aging study (n = 39, **left panel**). Statistical significance of co-clustering for same sex samples (low within and high between cluster distance) was performed using random permutation of factor levels between samples, red line shows observed values, P-values are shown as numbers near red lines (n = 10,000 permutations, **right panel**).
(TIF)

S5 Fig In-depth analysis of the cross-sample contamination issue. **A.** Example of three top clonotypes coming from different batches (A2, A3 and A4) clearly shows presence of intra-batch contamination. **B.** Frequency of parent clonotypes (x axis) and their contamination traces (y axis) in the pooled samples of aging study. Top 100 clonotypes having the largest frequency in pooled samples were analyzed. **C.** Input of cross-sample contamination to the observed inter-sample overlap (F measure) for samples coming from the same (red) and different (green) sequencing lane.
(TIF)

S6 Fig An example of Rep-Seq processing software comparison. Comparison of clonotype extraction efficiency on A4-i107 sample from the “aging” study described in S1 Text. Note that error correction in current case was performed using unique molecular identifiers, therefore this figure only deals with CDR3 mapping and clonotype assembly capabilities of software tools. MiTCR and MIGEC identified 95% and 98% of clonotypes found by IgBlast. False clonotype rate was 0.2% and 2.7% respectively.
(TIF)
