# Supplementary material for: VDJtools: Unifying Post-analysis of T Cell Receptor Repertoires
Source: PLoS Comput Biol. 2015 Nov 25;11(11):e1004503. doi: 10.1371/journal.pcbi.1004503 (PMC4659587; doi:10.1371/journal.pcbi.1004503)
Supplement: S1 Text — (PDF) [file pcbi.1004503.s001.pdf]

S1 Text. Description of dataset and benchmarks

Selecting an optimal measure for quantifying repertoire diversity

Diversity estimator benchmark (on a model of 39 healthy individuals from 6 to 90 years old dataset)

A great variety of diversity estimators are used in RepSeq studies (see literature overview in **S1 Table**). Among those are the observed diversity, Chao and Efron estimates on lower bound of total diversity, Shannon and Simpson diversity indices, the D50 measure (number of unique top-ranking clonotypes have a total frequency of 50% of profiled T-cells recent patent application) and extrapolated Chao estimate [51]. In order to perform a comprehensive benchmark of those measures, we have selected a recently published T-cell repertoire aging study [10] that provides both deep T-cell receptor beta chain sequencing data, donor age (physiological factor) and naïve T-cell count (basic immune status factor). Both these factors were shown to have a strong correlation with immune repertoire diversity. We have computed the Spearman R^2 coefficient for those two factors for each measure. Two benchmark settings were used: unmodified datasets and datasets down-sampled to the size of smallest dataset. Benchmark results are provided in **S1 Figure** with the best performance shown by Chao and Efron estimates on lower bound of total diversity in second setting.

Estimator biases (on a model of young MS patients and healthy donors dataset)

In order to quantify possible biases that affect repertoire diversity measures and select the optimal measure we have performed an ANOVA analysis for several diversity measures for the young MS patients and young healthy donors shown in **S3 Table**. We were particularly interested in how the sample **size** (here, the number T-cell receptor beta chain cDNA molecules or TRBM, see main text) affects diversity estimates, as it is intuitive that when rarefaction curves are far from saturation, as in **Figure 2** of the main text, deeper sampling could bias diversity estimates. We have also included the **age** factor based on our prior findings indicating that age has a substantial negative correlation with sample diversity [10].

We have chosen two commonly used diversity measures: observed diversity for samples normalized to the same size (**Observed, normalized**), the number of unique clonotypes in a fixed-size cDNA molecules subsample; and lower bound total diversity estimate, a measure computed based on clonotype frequencies, Chao 1 measure is used in current example (**Chao 1, total**). Those measures were used in Refs. [10] and [52].

Those measures both have potential drawbacks: while normalized observed diversity makes a limited use of the information of clonotype size distribution (sample clonality), lower bound total diversity estimate could be strongly biased by the sampling depth. We added an intuitive yet simple sample diversity measure, called normalized Chao 1 measure, which is lower bound total diversity estimate calculated based on repertoires down-sampled to the size of smallest sample

(**Chao 1, normalized**). A more complex diversity measure recently suggested by Colwell and colleagues [51] was also included. This measure is based on parametric extrapolation of observed diversity using Chao estimate for the number of unseen species (**Chao, normalized, extrapolated**).

ANOVA results (**S4 Table**) indicate that **Chao, total** diversity estimate at **S2 Figure** is biased, while both the **Chao, normalized** and **Chao, normalized, extrapolated** diversity were free of any bias yet were able to detect significant difference of repertoire diversity based on the **condition** factor (healthy/MS). The **Observed** diversity estimate was not sensitive enough to catch the effect, presumably due to the lack of information coming from clonotype size distribution.

Of note, the same conclusions could be obtained if using Efron-Thisted lower bound of total repertoire diversity estimate (not shown).

Optimizing parameters for repertoire clustering

Choosing repertoire similarity measure (on a model of three pairs of healthy identical twins datasets)

Several measures that describe how similar are two repertoires based on their clonotype composition are commonly used: number of unique overlapping clonotypes (d_{12}), Pearson correlation of overlapping clonotype frequencies (R), Jaccard and Morisita-Horn indices (see literature overview in **S1 Table**).

We propose an additional intuitive similarity measure *F*, which is computed as follows: given the total frequency (weight) of clonotypes overlapping between samples 1 and 2 is f_{12} in the first sample and f_{21} in the second sample, $F = \frac{1}{2} log_{10}(f_{12} f_{21})$.

Note that we are using normalized number of unique overlapping clonotypes, $D = \frac{d_{12}}{d_1 d_2}$,

where d_1 and d_2 are the numbers of unique clonotypes in individual samples, as suggested in Ref [14].

Choice of an appropriate benchmark example is a complex one. For example, comparing replicate blood samples drawn from the same individual to samples drawn from different individuals is a trivial test describing technical, but not biological variability. Comparing various T-cell subsets can be affected by limited accuracy of cell sorting. Here we have selected the data on identical twins' peripheral blood TCR alpha and beta repertoires reported in Ref. [54] as a immunologically relevant and challenging benchmark setting. Comparison of how various similarity measures can distinguish between TCR alpha and beta repertoires of identical twins and unrelated individuals is provided in **S5 Table** and **S3 Figure**. This comparison shows that only the F measure is significantly different between related twins and unrelated pairs for both TCR alpha and beta datasets.

Exploring biases in sample clustering (on a model of young MS patients and healthy donors datasets)

In this experimental design, TCR libraries were sample-barcoded at the 5'-end only. Amplified PCR products were joined before Illumina adapters ligation, and thus co-amplified after. According to our current experience, this cost-efficient approach is not well protected from the cross-sample contaminations resulting from the chimeric PCR products.

However, as CDR3 amino acid sequences could be encoded by several nucleotide variants, it is expected that for a robust biological phenomena involved T-cell receptor CDR3 sequences could still be traced on amino acid level, while not accounting for similar nucleotide CDR3 sequences that could arise due to cross-sample contaminations within the same batch. Therefore, we have compared CDR3 amino acid-non-nucleotide sequence matching rule for clonotype list intersection with commonly used CDR3 amino acid matching.

As could be seen from **S4A Figure**, the dendrogram for amino acid overlap rule was severely affected by batch effect in this experiment, which is not observed for amino acid-non-nucleotide matching rule. Note that amino acid-non-nucleotide overlap should be used with caution as it has far less power in detecting repertoire overlap comprised of clonotypes with low degree of convergent recombination and is not suitable for studying samples coming from the same individual. Protection from cross-sample contaminations within a batch can be provided by using double end sample barcoding within PCR and/or independent indexed Illumina adapters ligation (or PCR-incorporation) for each TCR library (data not shown).

As MS patients are female (this can be attributed to overall higher MS onset frequency in females) while the control set has both male and female samples, we have also checked for sex-specific sample clustering bias and have not detected any (**S4B Figure**).

To further quantify the extent of cross-sample contamination we have used three batches from the dataset containing 39 individuals of various ages (described above, see Diversity Estimator Benchmark section). The within-batch contamination is clearly distinguishable when looking at top clonotypes (**S5A Figure**), with the overall frequency of contaminant being higher for large clonotypes (**S5B Figure**). While the frequency of contaminating clones is ~1000 times less than their parent clonotypes, in summary they can account for ~10% sample overlap according to F measure described above, that may essentially influence estimations of the true inter-sample overlap.
