# Supplementary material for: VDJtools: Unifying Post-analysis of T Cell Receptor Repertoires
Source: PLoS Comput Biol. 2015 Nov 25;11(11):e1004503. doi: 10.1371/journal.pcbi.1004503 (PMC4659587; doi:10.1371/journal.pcbi.1004503)
Supplement: S1 Table — Primary analysis software and post-analysis methods that are supported by VDJtools are highlighted in green. Note that none of these papers indicate using specialized software for analysis of clonotype tables, therefore post-analysis in each case was performed either manually or using in-house scripts developed from a scratch. (DOCX) [file pcbi.1004503.s002.docx]

| Suppl.  Ref. | Technology | Primary analysis | Post-analysis | Details | Alternative VDJtools routine |
| --- | --- | --- | --- | --- | --- |
| 1 | iRepertoire | IMGT | Spectratype |  | CalcSpectratype |
|  |  |  | Segment usage |  | CalcSegmentUsage |
|  |  |  | Variable-Joining segment pairing |  | PlotFancyVJUsage |
|  |  |  | Clonality analysis | Top 100 clonotypes frequency distribution | QuantilePlot |
|  |  |  | Diversity estimates | Simpson index, Shannon index, D50 | CalcDiversityStats |
|  |  |  | Clonotype overlap | Venn diagram | JoinSamples |
| 2 | ImmunoSEQ | ImmunoSEQ | Clonotype expansion | Custom statistical test | FilterByAbundance |
| 3 | In-house | In-house | Variable-Joining segment pairing |  | PlotFancyVJUsage |
|  |  |  | Clonotype expansion | Clonotype frequency cutoff | FilterByAbundance |
|  |  |  | CDR3 amino acid composition |  | N/A |
| 4 | ImmunoSEQ | ImmunoSEQ | Diversity estimates | Simpson and Shannon indices | CalcDiversityStats |
|  |  |  | Clonotype expansion | Clonotype frequency cutoff | FilterByAbundance |
| 5 | ImmunoSEQ | ImmunoSEQ | Diversity estimates | Shannon index | CalcDiversityStats |
|  |  |  | Variable segment profile clustering |  | CalcSegementUsage |
|  |  |  | Segment usage |  | CalcSegmentUsage |
|  |  |  | CDR3 amino acid composition |  | N/A |
| 6 | iRepertoire | In-house | Spectratype |  | CalcSpectratype |
|  |  |  | Variable-Joining segment pairing |  | PlotFancyVJUsage |
|  |  |  | Clonality analysis |  | QuantilePlot |
| 7 | ImmunoSEQ | ImmunoSEQ | Diversity estimates | Reyni entropy | CalcDiversityStats |
|  |  |  | Clonotype overlap for a pair of samples |  | OverlapPair |
|  |  |  | Sample clustering | Jaccard index | CalcPairwiseDistances,  ClusterSamples |
| 8 | ImmunoSEQ | ImmunoSEQ | Segment usage |  | CalcSegmentUsage |
|  |  |  | Clonality analysis |  | QuantilePlot |
|  |  |  | Spectratype |  | CalcSpectratype |
|  |  |  | Overlap for a pair of samples |  | OverlapPair |
| 9 | In-house | MiTCR | Clonality analysis |  | QuantilePlot |
|  |  |  | Clonotype overlap for a pair of samples |  | OverlapPair |
|  |  |  | Diversity estimates | Observed diversity | CalcDiversityStats |
|  |  |  | Clonotype overlap | Venn diagram | JoinSamples |
| 10 | ImmunoSEQ | ImmunoSEQ | Clonality analysis |  | QuantilePlot |
|  |  |  | Clonotype overlap for a pair of samples |  | OverlapPair |
|  |  |  | Clonotype expansion | Frequency-based or custom statistical test | FilterByAbundance |
| 11 | In-house | IMGT | Clonotype overlap for a pair of samples |  | OverlapPair |
| 12 | In-house | In-house | Clonality analysis |  | QuantilePlot |
|  |  |  | Clonotype tracking |  | TrackClonotypes |
| 13 | In-house | In-house | Clonality analysis |  | QuantilePlot |
|  |  |  | CDR3 convergence |  | CalcBasicStats |
| 14 | In-house | IMGT | Variable-Joining segment pairing |  | PlotFancyVJUsage |
|  |  |  | Clonality analysis |  | QuantilePlot |
|  |  |  | Insert size distribution |  | CalcBasicStats |
|  |  |  | Non-coding clonotype frequency |  | CalcBasicStats |
|  |  |  | Diversity estimates |  | CalcDiversityStats |
|  |  |  | Clonotype overlap | Venn diagram | JoinSamples |
| 15 | ImmunoSEQ | ImmunoSEQ | Clonality analysis |  | QuantilePlot |
| 16 | In-house | IMGT | Diversity estimates | Shannon index, Simpson index, D50 | CalcDiversityStats |
|  |  |  | Segment usage |  | CalcSegmentUsage |
|  |  |  | Clonotype overlap | Venn diagram | JoinSamples |
| 17 | ImmunoSEQ | ImmunoSEQ | Clonality analysis |  | QuantilePlot |
| 18 | ImmunoSEQ | ImmunoSEQ | Clonal expansion | Clonotypes from top 25% qunatile | FilterByAbundance |
|  |  |  | Clonotype tracking |  | TrackClonotypes |
| 19 | In-house | In-house | Spectratype | (+detalization for top clonotypes) | CalcSpectratype |
|  |  |  | Segment usage |  | CalcSegmentUsage |
|  |  |  | Variable-Joining segment pairing |  | PlotFancyVJUsage |
|  |  |  | Clonotype overlap for a pair of samples |  | OverlapPair |
| 20 | iRepertoire | IMGT | Spectratype | (+detalization by Variable segment) | CalcSpectratype, PlotSpectratypeV |
|  |  |  | Clonality analysis |  | QuantilePlot |
|  |  |  | Segment usage |  | CalcSegmentUsage |

**S1 Table references**

1. Rechavi E, Lev A, Lee YN, Simon AJ, Yinon Y, Lipitz S, Amariglio N, Weisz B, Notarangelo LD, Somech R: **Timely and spatially regulated maturation of B and T cell repertoire during human fetal development.** *Sci Transl Med.* 2015 Feb **25;7(276):**276ra25.

2. DeWitt WS, Emerson RO, Lindau P, Vignali M, Snyder TM, Desmarais C, Sanders C, Utsugi H, Warren EH, McElrath J, Makar KW, Wald A, Robins HS: **Dynamics of the Cytotoxic T Cell Response to a Model of Acute Viral Infection.** *J Virol.* 2015 Feb 4. pii: JVI.03474-14.

3. Zhang Q, Jia Q, Deng T, Song B, Li L: **Heterogeneous expansion of CD4+ tumor-infiltrating T-lymphocytes in clear cell renal cell carcinomas.** *Biochem Biophys Res Commun.* 2015 Feb 27;**458(1):**70-6.

4. Morris H, DeWolf S, Robins H, Sprangers B, LoCascio SA, Shonts BA, Kawai T, Wong W, Yang S, Zuber J, Shen Y, Sykes M: **Tracking donor-reactive T cells: Evidence for clonal deletion in tolerant kidney transplant patients.** *Sci Transl Med.* 2015 Jan 28;**7(272):**272ra10.

5. Ramesh M, Hamm D, Simchoni N, Cunningham-Rundles C: **Clonal and constricted T cell repertoire in Common Variable Immune Deficiency.** *Clin Immunol.* 2015 Jan 14. pii: S1521-6616(15)00004-2.

6. Hunsucker SA, McGary CS, Vincent BG, Enyenihi AA, Waugh JP, McKinnon KP, Bixby LM, Ropp PA, Coghill JM, Wood WA, Gabriel DA, Sarantopoulos S, Shea TC, Serody JS, Alatrash G, Rodriguez-Cruz T, Lizée G, Buntzman AS, Frelinger JA, Glish GL, Armistead PM: **Peptide/MHC Tetramer-Based Sorting of CD8+ T Cells to a Leukemia Antigen Yields Clonotypes Drawn Nonspecifically from an Underlying Restricted Repertoire.** *Cancer Immunol Res.* 2015 Jan 9.

7. Spreafico R, Rossetti M, van Loosdregt J, Wallace CA, Massa M, Magni-Manzoni S, Gattorno M, Martini A, Lovell DJ, Albani S: **A circulating reservoir of pathogenic-like CD4+ T cells shares a genetic and phenotypic signature with the inflamed synovial micro-environment.** *Ann Rheum Dis.* 2014 Dec 12. pii: annrheumdis-2014-206226.

8. Toivonen R, Arstila TP, Hänninen A: **Islet-associated T-cell receptor-β CDR sequence repertoire in prediabetic NOD mice reveals antigen-driven T-cell expansion and shared usage of VβJβ TCR chains.** *Mol Immunol.* 2015 Mar;**64(1):**127-35.

9. Becattini S, Latorre D, Mele F, Foglierini M, De Gregorio C, Cassotta A, Fernandez B, Kelderman S, Schumacher TN, Corti D, Lanzavecchia A, Sallusto F: **T cell immunity. Functional heterogeneity of human memory CD4⁺ T cell clones primed by pathogens or vaccines.** *Science.* 2015 Jan 23;**347(6220):**400-6.

10. Tumeh PC, Harview CL, Yearley JH, Shintaku IP, Taylor EJ, Robert L, Chmielowski B, Spasic M, Henry G, Ciobanu V, West AN, Carmona M, Kivork C, Seja E, Cherry G, Gutierrez AJ, Grogan TR, Mateus C, Tomasic G, Glaspy JA, Emerson RO, Robins H, Pierce RH, Elashoff DA, Robert C, Ribas A: **PD-1 blockade induces responses by inhibiting adaptive immune resistance.** *Nature.* 2014 Nov 27;**515(7528):**568-71.

11. Emerson RO, Mathew JM, Konieczna IM, Robins HS, Leventhal JR: **Defining the alloreactive T cell repertoire using high-throughput sequencing of mixed lymphocyte reaction culture.** *PLoS One.* 2014 Nov 3;**9(11):**e111943.

12. Remmerswaal EB, Klarenbeek PL, Alves NL, Doorenspleet ME, van Schaik BD, Esveldt RE, Idu MM, van Leeuwen EM, van der Bom-Baylon N, van Kampen AH, Koch SD, Pircher H, Bemelman FJ, Ten Brinke A, Baas F, Ten Berge IJ, van Lier RA, de Vries N: **Clonal evolution of CD8+ T cell responses against latent viruses: relationship among phenotype, localization, and function.** *J Virol.* 2015 Jan;**89(1):**568-80.

13. van Schaik B, Klarenbeek P, Doorenspleet M, van Kampen A, Moody DB, de Vries N, Van Rhijn I: **Discovery of invariant T cells by next-generation sequencing of the human TCR α-chain repertoire.** *J Immunol.* 2014 Nov 15;193(10):5338-44.

14. Li Z, Long M, ChunMei L, Bin S, Jiang Y, Rui M, Qingqing M, XinSheng Y: **Composition and variation analysis of TCR β-chain CDR3 repertoire in the thymus and spleen of MRL/lpr mouse at different ages.** *Immunogenetics.* 2015 Jan;**67(1):**25-37.

15. Rajala HL, Olson T, Clemente MJ, Lagström S, Ellonen P, Lundan T, Hamm DE, Zaman SA, Lopez Marti JM, Andersson EI, Jerez A, Porkka K, Maciejewski JP, Loughran TP, Mustjoki S: **The analysis of clonal diversity and therapy responses using STAT3 mutations as a molecular marker in large granular lymphocytic leukemia.** *Haematologica.* 2015 Jan;**100(1):**91-9.

16. Wu J, Liu D, Tu W, Song W, Zhao X: **T-cell receptor diversity is selectively skewed in T-cell populations of patients with Wiskott-Aldrich syndrome.** *J Allergy Clin Immunol.* 2015 Jan;**135(1):**209-16.

17. Dudgeon C, Chan C, Kang W, Sun Y, Emerson R, Robins H, Levine AJ: **The evolution of thymic lymphomas in p53 knockout mice.** *Genes Dev.* 2014 Dec 1;**28(23):**2613-20.

18. Bajor DL, Xu X, Torigian DA, Mick R, Garcia LR, Richman LP, Desmarais C, Nathanson KL, Schuchter LM, Kalos M, Vonderheide RH: **Immune activation and a 9-year ongoing complete remission following CD40 antibody therapy and metastasectomy in a patient with metastatic melanoma.** *Cancer Immunol Res.* 2014 Nov;**2(11):**1051-8.

19. Li D, Gao G, Li Z, Sun W, Li X, Chen N, Sun J, Yang Y: **Profiling the T-cell receptor repertoire of patient with pleural tuberculosis by high-throughputsequencing.** *Immunol Lett.* 2014 Nov;**162(1 Pt A):**170-80.

20. O'Connell AE, Volpi S, Dobbs K, Fiorini C, Tsitsikov E, de Boer H, Barlan IB, Despotovic JM, Espinosa-Rosales FJ, Hanson IC, Kanariou MG, Martínez-Beckerat R, Mayorga-Sirera A, Mejia-Carvajal C, Radwan N, Weiss AR, Pai SY, Lee YN, Notarangelo LD: **Next generation sequencing reveals skewing of the T and B cell receptor repertoires in patients with Wiskott-Aldrich syndrome.** *Front Immunol.* 2014 Jul 18;**5:**340.
