# Supplementary material for: VDJtools: Unifying Post-analysis of T Cell Receptor Repertoires
Source: PLoS Comput Biol. 2015 Nov 25;11(11):e1004503. doi: 10.1371/journal.pcbi.1004503 (PMC4659587; doi:10.1371/journal.pcbi.1004503)
Supplement: S2 Table — This table contains summary of features present in VDJtools and other immune repertoire post-analysis software (ImmunoSEQ analyzer [38], Vidjill [7] and AbMining Toolbox [39]). (DOCX) [file pcbi.1004503.s003.docx]

| Features | VDJtools | ImmunoSEQ Analyzer | Vidjill | AbMining Toolbox |
| --- | --- | --- | --- | --- |
| **Basic analysis** | | | | |
| Segment usage | + | + | + | - |
| - V/J pairing | + | + | + | - |
| Spectratyping | + | + | - | + |
| - detailed | + | - | - | - |
| - by segment | + | + | - | - |
| **Repertoire diversity analysis** | | | | |
| Diversity estimation | + | - | - | - |
| Clonality (clonotype frequency distribution) | + | + | + | + |
| **Clonotype overlap** | | | | |
| Overlap, sample pair (scatterplot, etc) | + | + | - | + |
| Repertoire similarity estimation | + | partial (up to 25 samples, correlation only) | - | - |
| Repertoire clustering | + | - | - | - |
| Clonotype tracking | + | + | + | - |
| Overlap, set of samples (Venn diagram, etc) | + | + | - | - |
| **Operations on clonotype tables** | | | | |
| Pool samples | + | - | - | - |
| Join samples | + | - | - | - |
| Down-sampling | + | - | - | + |
| **Clonotype filtering** | | | | |
| Basic filtering (by segment, non-coding, frequency) | + | + | + | - |
| Cross-sample contamination filtering and filtering against a sample | + | - | - | - |
| **Other** | | | | |
| Sequence detail (non-coding, VDJ markup) | partial (with VDJviz) | + | - | + |
| Sequence amino acid composition analysis | - | - | - | + |
| Somatic hypermutation analysis | - | + | - | - |
| Database search (known CDR3) | + | - | - | - |
| Clonotype browsing (GUI) | + (VDJviz) | + | + | - |
| Supported formats | | | | |
|  | VDJtools, ImmunoSEQ, IMGT, MiGEC, MiTCR, IgBlast | ImmunoSEQ | Vidjill | AbMining Toolbox |
| Software distributive type and licence | | | | |
|  | Standalone, web server (VDJviz) | Web server | Web server | Standalone |
|  | Open-source | Commercial | Open-source | Open-source |
