# Supplementary material for: VDJtools: Unifying Post-analysis of T Cell Receptor Repertoires
Source: PLoS Comput Biol. 2015 Nov 25;11(11):e1004503. doi: 10.1371/journal.pcbi.1004503 (PMC4659587; doi:10.1371/journal.pcbi.1004503)
Supplement: S3 Table — Metadata for MS and control samples. Donor age, gender and condition are provided. Samples in the same batch were prepared together, multiplexed and sequenced on the same HiSeq lane. (DOCX) [file pcbi.1004503.s004.docx]

| Sample id | Age, y | Gender | Condition* | Study | Batch | Read count | cDNA count |
| --- | --- | --- | --- | --- | --- | --- | --- |
| C1 | 11 | M | Healthy | [[10]](#h.4d34og8) | A | 6177177 | 1024462 |
| C2 | 9 | M | Healthy | [[10]](#h.4d34og8) | A | 7557527 | 901741 |
| C3 | 6 | F | Healthy | [[10]](#h.4d34og8) | A | 6389442 | 1307129 |
| C4 | 16 | M | Healthy | [[10]](#h.4d34og8) | A | 6278235 | 1025212 |
| C5 | 16 | M | Healthy | [[10]](#h.4d34og8) | A | 4794896 | 985703 |
| C6 | 10 | M | Healthy | [[10]](#h.4d34og8) | A | 5389823 | 969668 |
| C7 | 25 | M | Healthy | [[10]](#h.4d34og8) | B | 6444467 | 1349243 |
| C8 | 22 | F | Healthy | [[10]](#h.4d34og8) | B | 7003948 | 847147 |
| C9 | 24 | F | Healthy | [[10]](#h.4d34og8) | B | 6735421 | 1378057 |
| C10 | 20 | M | Healthy | [[10]](#h.4d34og8) | B | 4468360 | 244961 |
| C11 | 21 | F | Healthy | [[10]](#h.4d34og8) | B | 6019628 | 596196 |
| MS1** | 12 | F | MS-II | present | C | 2082189 | 457905 |
| MS2 | NA | NA | MS | present | C | 2033428 | 217436 |
| MS3 | 8 | F | MS-IV | present | C | 2053790 | 370660 |
| MS4 | 14 | F | MS-II | present | C | 1645726 | 162964 |
| MS5 | 15 | F | MS-I | present | C | 1928054 | 420918 |
| MS6 | 15 | F | MS-IV | present | C | 1885385 | 390649 |
| MS7 | NA | NA | MS | present | C | 2732824 | 590545 |
| MS8 | 14 | F | MS | present | C | 2615142 | 555428 |
| MS9 | 6 | F | MS | present | C | 2177280 | 492419 |
| MS10 | 15 | F | MS | present | C | 3549929 | 720586 |
| MS11 | 14 | F | MS-IV | present | C | 2264440 | 426057 |
| MS12 | 13 | F | MS-IV | present | C | 3795039 | 366527 |
| MS13 | 14 | F | MS-IV | present | C | 3842968 | 246902 |
| MS8-HSCT*** | 14 | F | MS->HSCT | present | C | 2625856 | 597930 |

* MS - multiple sclerosis, MS-I - relapsing-remitting multiple sclerosis, MS-II - secondary progressive multiple sclerosis, MS-IV - progressive-relapsing multiple sclerosis

** acute phase of multiple sclerosis was observed in clinical setting

*** MS8-HSCT is the second blood draw from MS8 patient after undergoing a hematopoietic stem cell transplantation (HSCT). Note that the corresponding clonotype table file is called MS14, this name is also used in all pipeline scripts.
