# Supplementary material for: VDJtools: Unifying Post-analysis of T Cell Receptor Repertoires
Source: PLoS Comput Biol. 2015 Nov 25;11(11):e1004503. doi: 10.1371/journal.pcbi.1004503 (PMC4659587; doi:10.1371/journal.pcbi.1004503)
Supplement: S4 Table — Interaction between factors is shown with “:” sign. (DOCX) [file pcbi.1004503.s005.docx]

| Factor(s) | Chao 1,  total | Chao 1,  normalized | Chao, normalized,  extrapolated | Observed,  normalized |
| --- | --- | --- | --- | --- |
| age | 0.5, ns | 0.7, ns | 0.8, ns | 0.4, ns |
| condition | **0.002, **** | **0.02, *** | **0.02, *** | 0.7, ns |
| size | **0.03, *** | 0.2, ns | 0.15, ns | 0.4, ns |
| age:condition | 0.8, ns | 0.9, ns | 0.68, ns | 0.1, ns |
| age:size | **0.04, *** | 0.06, ns | 0.07, ns | 0.2, ns |
| state:size | 0.4, ns | 0.4, ns | 0.1, ns | 0.9, ns |
| age:condition:size | 0.2, ns | 0.2, ns | 0.2, ns | 0.07, ns |
