# Supplementary material for: VDJtools: Unifying Post-analysis of T Cell Receptor Repertoires
Source: PLoS Comput Biol. 2015 Nov 25;11(11):e1004503. doi: 10.1371/journal.pcbi.1004503 (PMC4659587; doi:10.1371/journal.pcbi.1004503)
Supplement: S5 Table — The ability of repertoire similarity measures to distinguish identical twins (n = 3 pairs) from unrelated individuals (n = 12) for TCR alpha and beta chain samples. Statistical significance and effect size were assessed using two-tailed T-test P-values and Cohen’s d. (DOCX) [file pcbi.1004503.s006.docx]

| TCR dataset | Similarity measure | P-value | Cohen’s d |
| --- | --- | --- | --- |
| alpha | Pearson correlation (R) | 0.18 | 0.48 |
|  | Normalized number of clonotypes (D) | 0.66 | 0.60 |
|  | Normalized frequency (F) | 0.008 | 1.80 |
|  | Jaccard index | 0.23 | 0.91 |
|  | Morisita-horn index | 0.20 | 0.50 |
| beta | Pearson correlation (R) | 0.13 | 0.51 |
|  | Normalized number of clonotypes (D) | 0.56 | 0.81 |
|  | Normalized frequency (F) | 0.03 | 1.47 |
|  | Jaccard index | 0.41 | 0.70 |
|  | Morisita-horn index | 0.10 | 0.56 |
