# Supplementary material for: VDJtools: Unifying Post-analysis of T Cell Receptor Repertoires
Source: PLoS Comput Biol. 2015 Nov 25;11(11):e1004503. doi: 10.1371/journal.pcbi.1004503 (PMC4659587; doi:10.1371/journal.pcbi.1004503)
Supplement: S6 Table — In order to determine the possible Type I-II bias according to Ref. [62], i.e. sharing of common repertoire features under the absence of common clonotypes, in TCR repertoires of MS patients we have performed multiple testing for TRBV frequency difference using one-tailed T-test. One-tailed T-test was chosen to increase the power as we a priori search for an expansion in the T-cell compartment. Appropriate correction for multiple testing was applied (Benjamini-Hockberg correction). Variable segments that are significantly over-represented in MS samples comparing to control are shown. (DOCX) [file pcbi.1004503.s007.docx]

| Variable segment | Fold-change | P-value* | Known? |
| --- | --- | --- | --- |
| TRBV5-6 | 1.6 | 2x10^-5^ | [61] |
| TRBV5-1 | 1.5 | 5x10^-4^ | [63] |
| TRBV7-6 | 1.4 | 2x10^-2^ | - |
| TRBV5-8 | 1.3 | 4x10^-2^ | - |
| TRBV20-1 | 1.3 | 2x10^-3^ | [[64]*](#h.lnxbz9)* |

* Benjamini-Hochberg corrected P-values from one-sided T-test

** Controversial evidence
