# Supplementary figures and images for: VDJtools: Unifying Post-analysis of T Cell Receptor Repertoires
Source: PLoS Comput Biol. 2015 Nov 25;11(11):e1004503. doi: 10.1371/journal.pcbi.1004503 (PMC4659587; doi:10.1371/journal.pcbi.1004503)

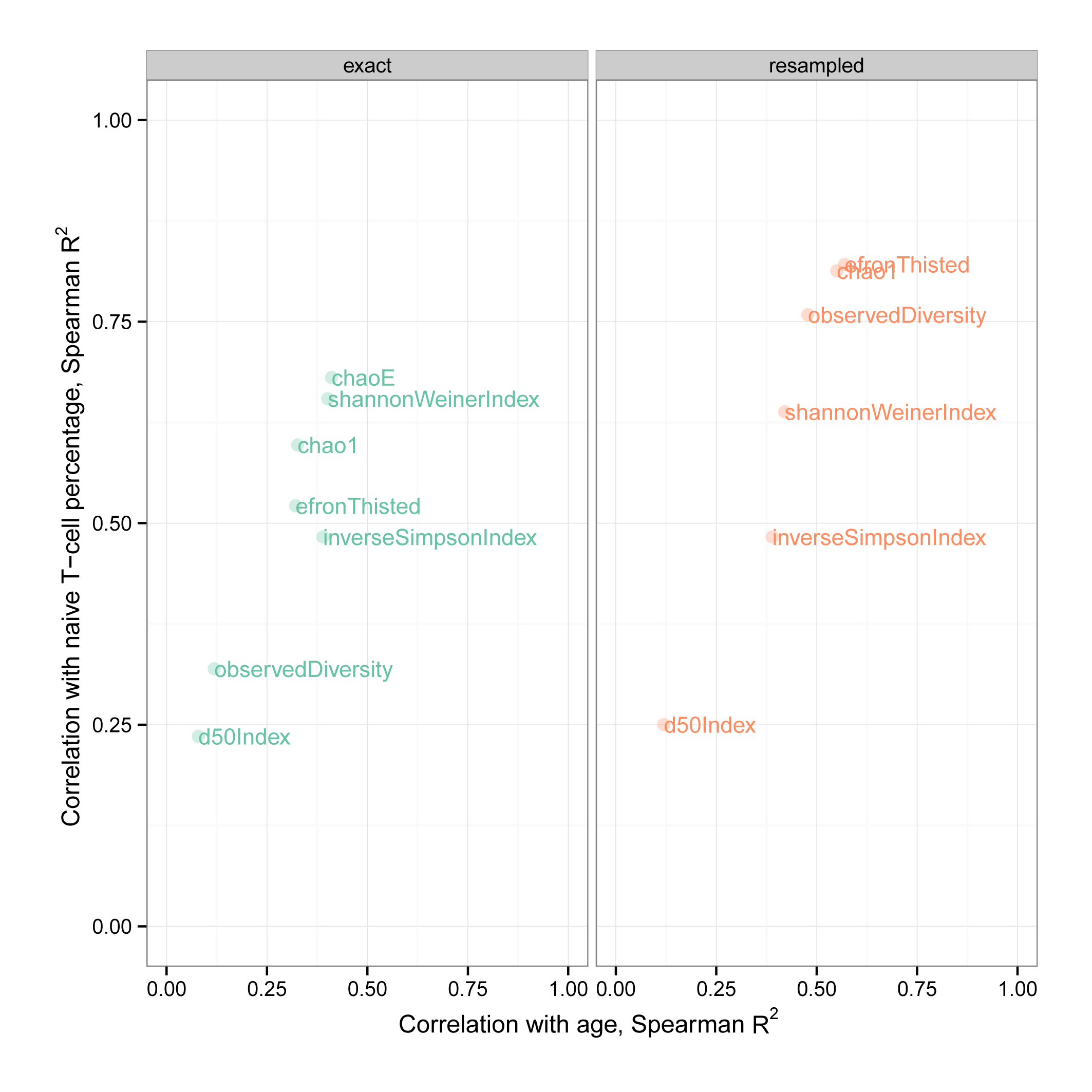

Supplement: S1 Fig — This plot shows Spearman correlation of diversity estimate with age and naïve T-cell count. Unmodified samples (exact) and samples normalized to the same size (resampled) from the “aging” study were used (n = 39). Note that ChaoE is omitted from the “resampled” plot, as it equals observed diversity when samples are of the same size. (TIF) [file pcbi.1004503.s008.tif]

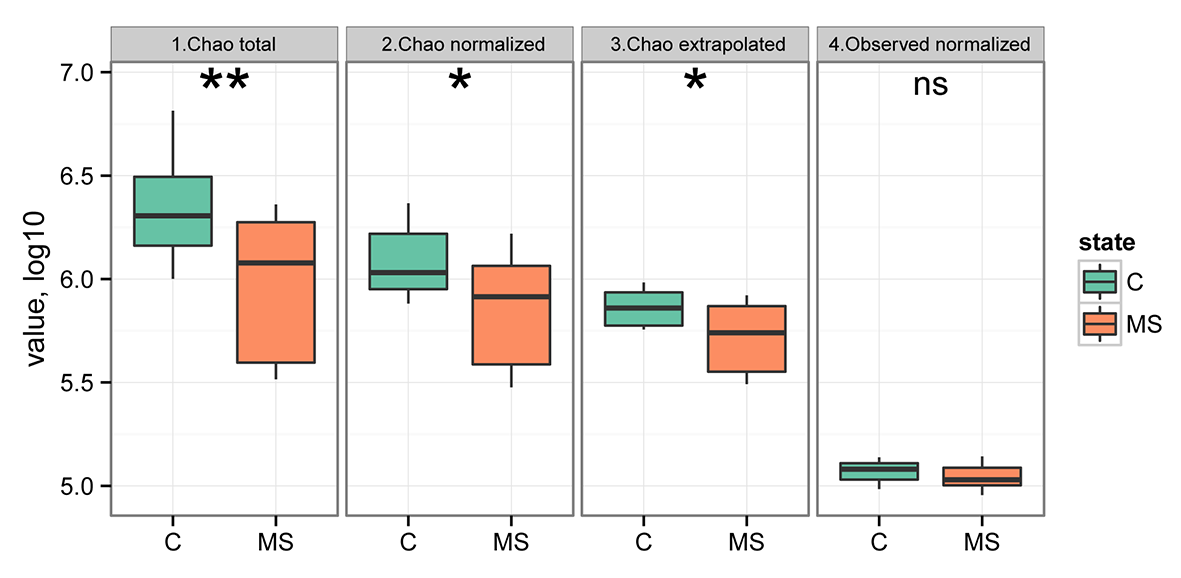

Supplement: S2 Fig — Difference was measured using four repertoire diversity estimates considered in present study (separate panels). The effect sizes are 1.21, 0.98, 0.95 and 0.46 respectively (Cohen’s d). **—P < 0.01, *—P < 0.05, ns—non-significant, two-tailed T-test. (TIF) [file pcbi.1004503.s009.tif]

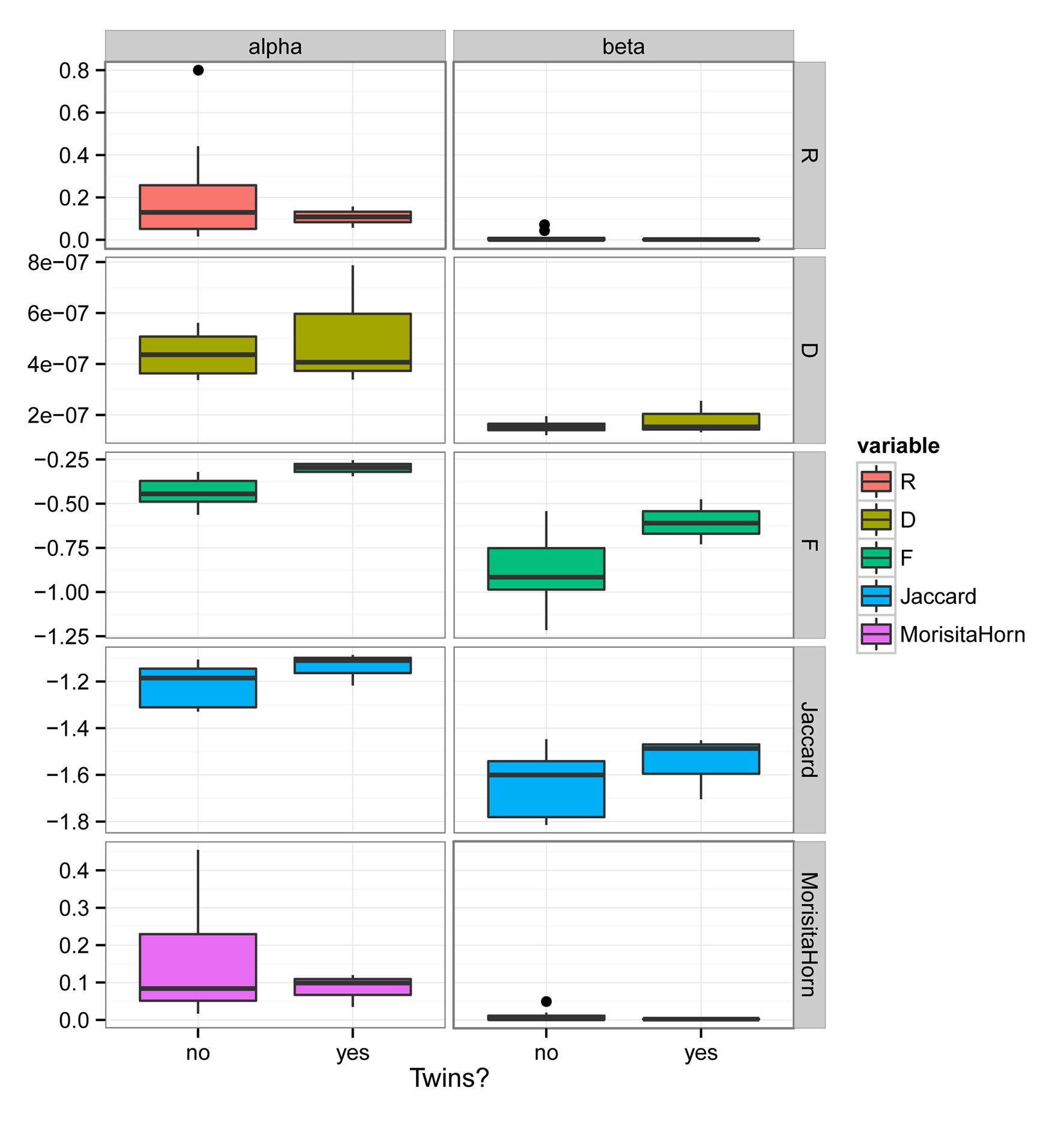

Supplement: S3 Fig — Values of similarity measures for identical twins and unrelated individuals that were used for statistical testing in S5 Table. (TIF) [file pcbi.1004503.s010.tif]

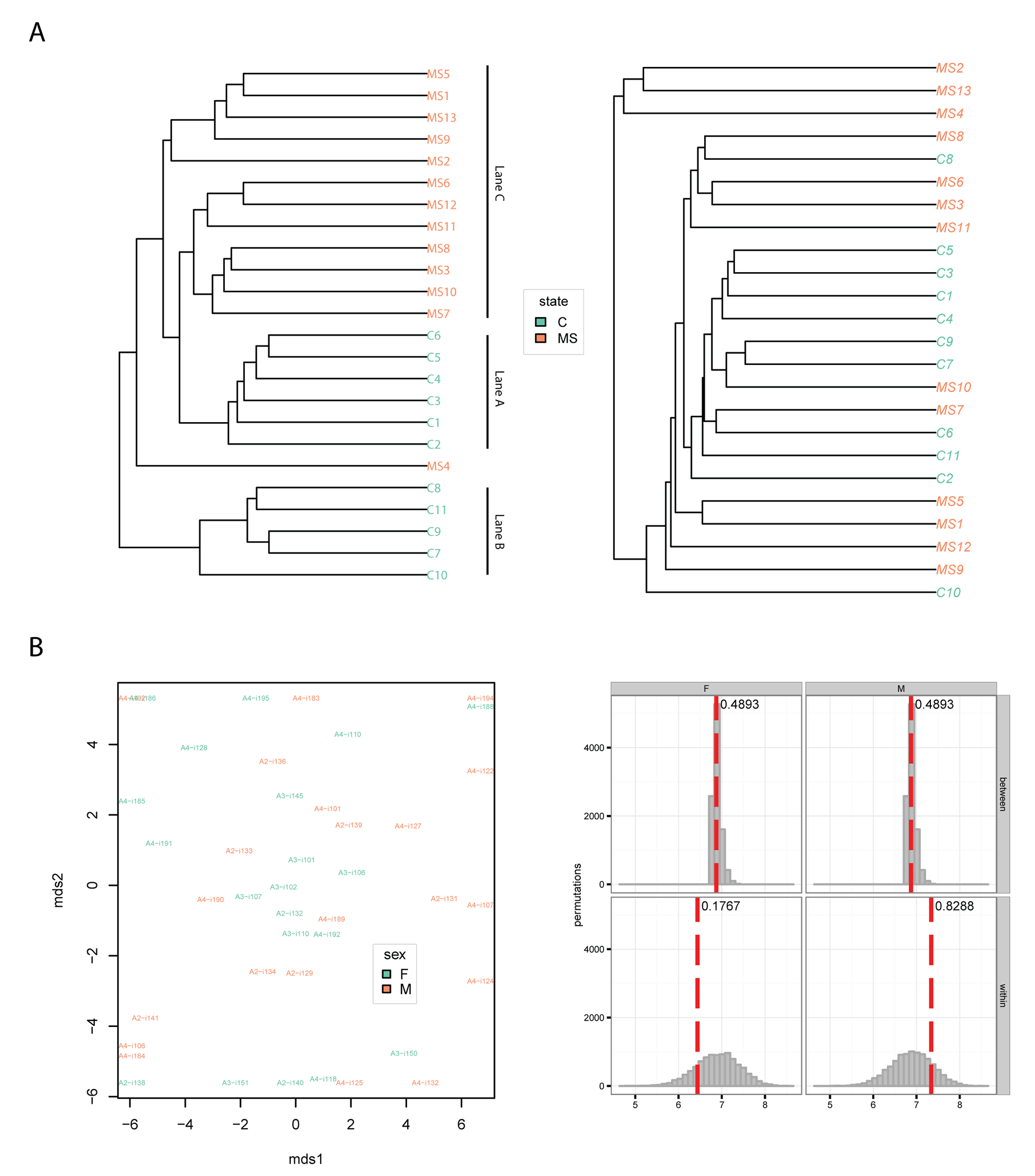

Supplement: S4 Fig — A. Hierarchical clustering of repertoires based on two distinct clonotype matching rules: matching CDR3 amino-acid sequences (left panel) and matching of CDR3 amino acid sequences but distinct CDR3 nucleotide sequences (right panel). Batch effect for samples on the same sequencing lane is shown with vertical lines. B. Checking for possible sex bias in repertoire clustering. Multi-dimensional scaling (MDS) plot is shown for healthy donors of various ages and sexes from the aging study (n = 39, left panel). Statistical significance of co-clustering for same sex samples (low within and high between cluster distance) was performed using random permutation of factor levels between samples, red line shows observed values, P-values are shown as numbers near red lines (n = 10,000 permutations, right panel). (TIF) [file pcbi.1004503.s011.tif]

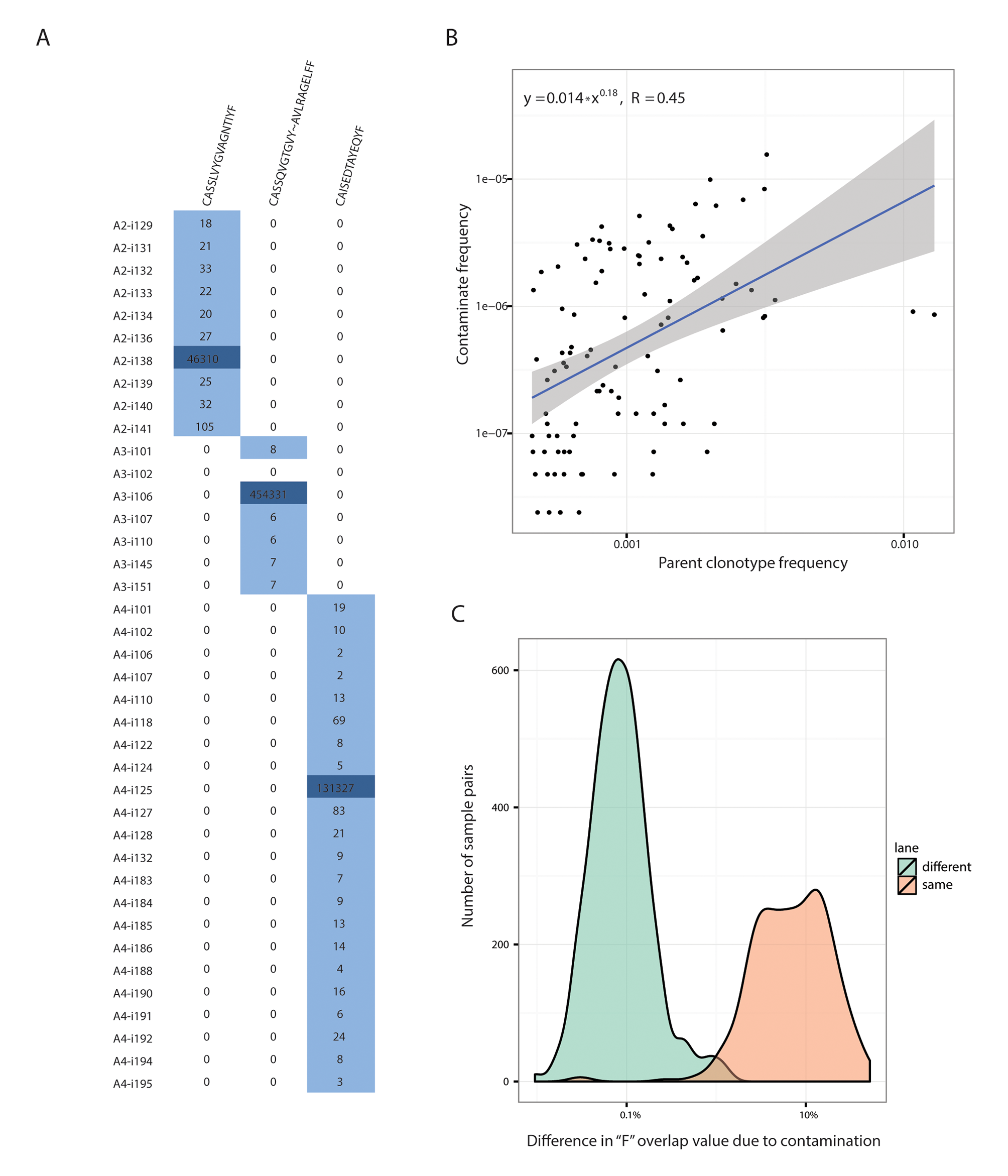

Supplement: S5 Fig — A. Example of three top clonotypes coming from different batches (A2, A3 and A4) clearly shows presence of intra-batch contamination. B. Frequency of parent clonotypes (x axis) and their contamination traces (y axis) in the pooled samples of aging study. Top 100 clonotypes having the largest frequency in pooled samples were analyzed. C. Input of cross-sample contamination to the observed inter-sample overlap (F measure) for samples coming from the same (red) and different (green) sequencing lane. (TIF) [file pcbi.1004503.s012.tif]

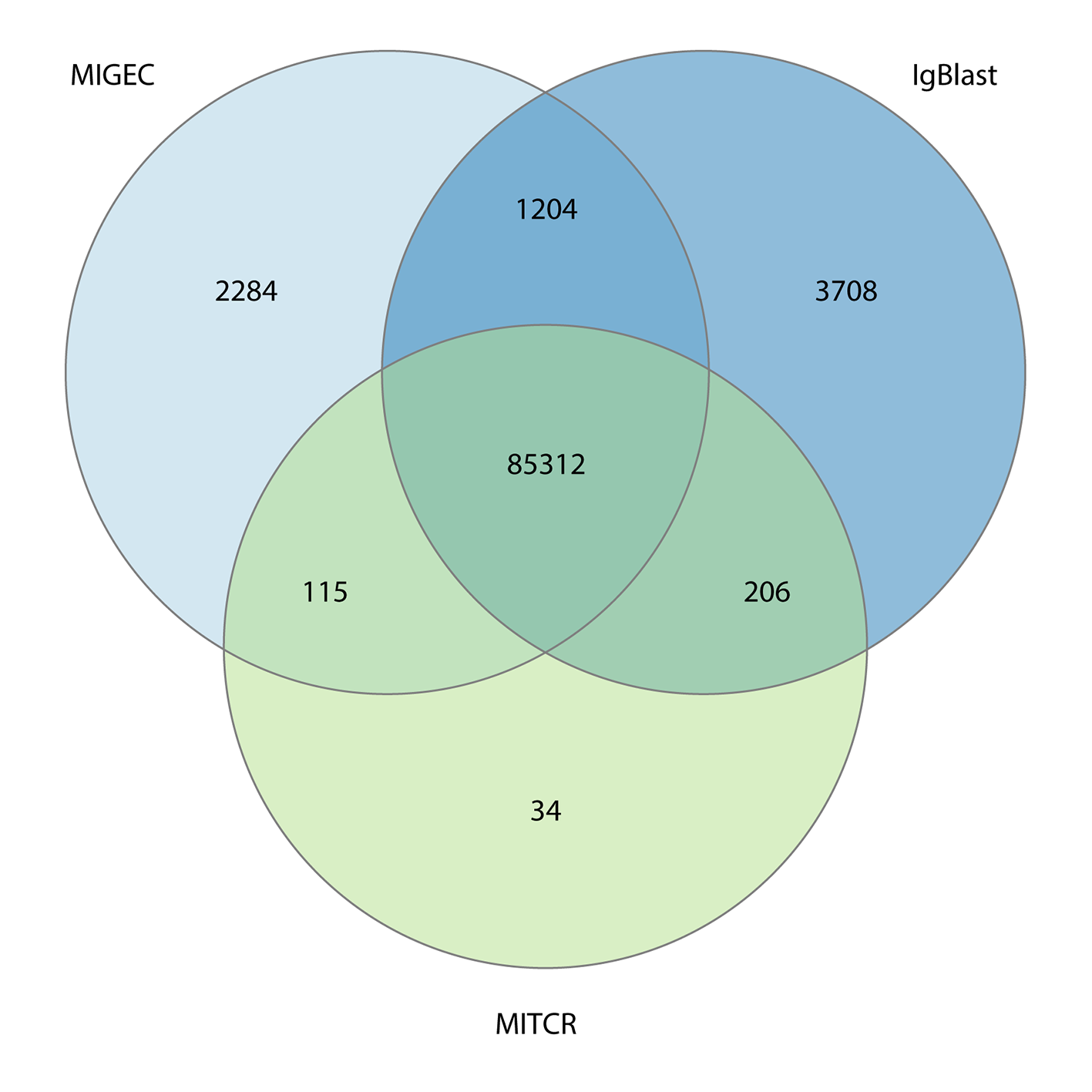

Supplement: S6 Fig — Comparison of clonotype extraction efficiency on A4-i107 sample from the “aging” study described in S1 Text. Note that error correction in current case was performed using unique molecular identifiers, therefore this figure only deals with CDR3 mapping and clonotype assembly capabilities of software tools. MiTCR and MIGEC identified 95% and 98% of clonotypes found by IgBlast. False clonotype rate was 0.2% and 2.7% respectively. (TIF) [file pcbi.1004503.s013.tif]
